# Agarose, Alginate and Chitosan Nanostructured Aerogels for Pharmaceutical Applications: A Short Review

**DOI:** 10.3389/fbioe.2021.688477

**Published:** 2021-05-12

**Authors:** Mariangela Guastaferro, Ernesto Reverchon, Lucia Baldino

**Affiliations:** Department of Industrial Engineering, University of Salerno, Fisciano, Italy

**Keywords:** agarose, alginate, chitosan, aerogel, supercritical CO_2_, drug delivery

## Abstract

In this short review, drug delivery systems, formed by polysaccharide-based (i.e., agarose, alginate, and chitosan) aerogels, are analyzed. In particular, the main papers, published in the period 2011–2020 in this research field, have been investigated and critically discussed, in order to highlight strengths and weaknesses of the traditional production techniques (e.g., freeze-drying and air evaporation) of bio-aerogels with respect to supercritical CO_2_ assisted drying. Supercritical CO_2_ assisted drying demonstrated to be a promising technique to produce nanostructured bio-aerogels that maintain the starting gel volume and shape, when the solvent removal occurs at negligible surface tension. This characteristic, coupled with the possibility of removing also cross-linking agent residues from the aerogels, makes these advanced devices safe and suitable as carriers for controlled drug delivery applications.

## Introduction

Pharmaceutical industry is evolving from traditional drug delivery systems, in which a biopolymeric matrix is used to provide weight, volume and flowability, toward new formulations, in which a biopolymer is adopted as drug performance enhancer in terms of release time and bioavailability (Agüero et al., 2017; Yuan et al., 2018; Shi et al., 2019; Wei et al., 2020; Liu et al., 2021).

In this field, the production of micro- and nanoparticles has been widely investigated, since they provide effective ways to address issues related to poorly water-soluble drugs and patient compliance (Agnihotri et al., 2004; Markman et al., 2013; Singh et al., 2019; Guastaferro et al., 2020).

Nowadays, also polymeric gels are becoming promising matrices for drug delivery, thanks to their nanostructured morphology that allows to reach larger drug loadings and an improved controlled release of the active compounds over time. In this regard, Cardea et al. (2018) realized poly(vinylidene fluoride-hexafluoropropylene) (PVDF-HFP) aerogels loaded with curcumin in order to obtain a prolonged drug release. Nanofibrous aerogels characterized by open interconnected pores and high porosity value (almost 94%) were produced by supercritical drying, and curcumin release was extended up to 44 h. Also Follmann et al. (2020) realized nanofibrous silica-based hybrid gels, with the aim to deliver camptothecin (CPT), a poorly water-soluble anticancer drug, in a sustained manner. In this case, aerogels ensured CPT release for more than 2 weeks. Giray et al. (2012) realized a composite gel consisting of a silica core coated by polyethyleneglycol (PEG). The results indicated that a slower release of ketoprofen was achieved increasing PEG diacrylate concentration, since it lowered aerogel permeability when it was immersed in an aqueous solution. Therefore, the main advantages of these systems over the traditional ones are: (i) tendency to deliver pharmaceutical compounds more selectively to a specific site, (ii) to maintain drug levels in the desired range, (iii) to increase patient compliance, and (iv) to prevent side effects (Van der Lubben et al., 2001; Guenther et al., 2008; Mehling et al., 2009; García-González et al., 2011; Marin et al., 2014; Lovskaya et al., 2015; Sosnik et al., 2021).

In this context, biocompatibility and biodegradability are essential features; therefore, polysaccharide-based polymers can be considered as “key formulation ingredients,” due to their natural properties (Zheng et al., 2015; Ferreira et al., 2016; Manivasagan and Oh, 2016; Wang et al., 2019). However, the final porous structure of these gels, required for drug release, depends on the kind of drying technique used. In particular, gels can be termed as “xerogel” when sample drying is carried out under ambient pressure and at room temperature, for some days (Conzatti et al., 2017; Takeshita et al., 2020b). Despite the energy-saving advantage of this technique, it leads to the formation of a condense structure that may have low porosity values and large shrinkage (Mirzaei et al., 2013; Buchtová and Budtova, 2016; Sukhodub et al., 2018; López-Iglesias et al., 2019). “Cryogels” are produced when the solvent inside the gel matrix is extracted by freeze drying. During this process, the liquid part in the wet-gel is frozen and, subsequently, under low pressure, the frozen wet-gel is dried by sublimation (Cheng et al., 2012; Gupta and Nayak, 2016; Mahmoud and Salama, 2016; You et al., 2017; Rubio-Elizalde et al., 2019; Yang et al., 2019).

**FIGURE 1:**
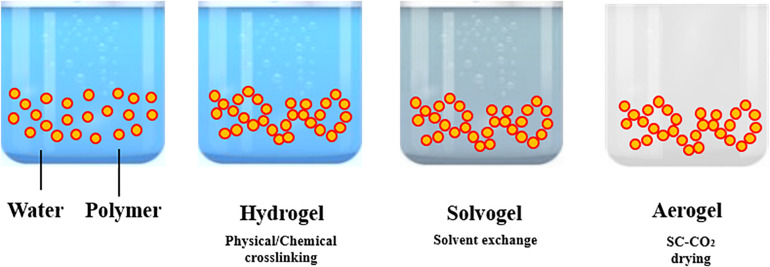
Aerogel production procedure.

The processing steps involved in “aerogels” production are summarized in Figure 1. Aerogel production frequently starts from the formation of a gel in an aqueous solution, adding a chemical, physical or enzymatic cross-linker. Operating in this way, a hydrogel is obtained. The following step is the replacement of the water present in the 3-D network of the hydrogel by an organic solvent; the resulting gel is named as solvogel. Solvent exchange step is a critical and dynamic process that can strongly affect the final aerogel morphology. In particular, solvent composition, kind of solvent and exchange rate are the main parameters to be investigated (Takeshita et al., 2020a). According to Takeshita et al. (2020a), a low affinity between the polymer forming the gel and the substituting solvent induces the formation of an aerogel-like structure during the solvent exchange itself and a drastic shrinkage of the gel. Therefore, to minimize this phenomenon, some guidelines can be followed: (i) to select an organic solvent at high affinity with the biopolymer (Takeshita et al., 2020a); (ii) to perform a multi-step solvent exchange (García-González et al., 2011; Baldino et al., 2019), at increasing percentage by volume of the solvent substituting water. Operating in this way, the liquid-liquid extraction and substitution of water with the organic solvent selected, evolves gradually, reducing gel shrinkage and other undesired structure modifications. Then, the solvent is extracted from the solid network by supercritical CO_2_ (SC-CO_2_): a supercritical mixture with an almost zero surface tension is formed, at the opportune operative conditions of pressure and temperature, between CO_2_ and the organic solvent that avoids the collapse of the delicate gel nanostructure (Reverchon et al., 2008; Cardea et al., 2009; Baldino et al., 2016; Muñoz-Ruíz et al., 2019).

Gels dried under supercritical conditions show unique properties, such as large porosity, uniform pore sizes, and high surface area, in the range of 500–1,200 m^2^/g, due to mesoporous (<50 nm and >2 nm) and micropores (<2 nm) distribution inside the polymeric matrix (Soleimani Dorcheh and Abbasi, 2008; Baldino et al., 2015, 2019; García-González et al., 2015). Moreover, they are made up of about 95% of air or gas by volume and, consequently, are very light in weight (Del Gaudio et al., 2013; Della Porta et al., 2013; Lu et al., 2014; Mallepally et al., 2015; Quraishi et al., 2015; Baldino et al., 2019). Aerogels could overcome the problems associated with slow drug dissolution rate, unfavorable pharmacokinetics, poor bio-distribution and lack of selectivity for target tissues (Ulker and Erkey, 2014; Lovskaya et al., 2015; Mohammadian et al., 2018). Therefore, the combination of the outstanding structural properties of aerogels with the physiological compatibility of polysaccharides would result in high potential drug delivery systems (Huang et al., 2011; Matricardi et al., 2013; Shelke et al., 2014; Ren et al., 2018).

In this short review, the attention will be focused on the production of chitosan (CS), alginate (ALG), and agarose (AGR) aerogels for the pharmaceutical field. Indeed, among the other applications, these biopolymers have also been studied for drug delivery, and the respective percentages of investigation are: 11.92% agarose, 37.74% alginate and 50.34% chitosan. They were calculated using the database Science Direct, looking at the number of papers written in the period 2011–2020. The path used was the following one: “biopolymer (AGR/ALG/CS), drug delivery, aerogel/cryogel.” Strengths and weaknesses of the traditional production techniques (e.g., freeze-drying and air evaporation) of these bio-aerogels will be critically compared with supercritical CO_2_ assisted drying, to highlight possible indications to obtain advanced bio-carriers for controlled drug delivery applications.

## Chitosan-Based Gels

Chitosan is derived from chitin that is the major component of the crustacean exoskeleton, and is naturally hydrophilic. CS exhibits biocompatible, biodegradable, and non-toxic properties (Pillai et al., 2009; Venkatesan and Kim, 2010).

The first step toward CS-based aerogel production is represented by hydrogel formation (Shi et al., 2021). CS hydrogels can be prepared by non-covalent strategies that take advantage of ionic interactions, H-bonding and Van der Waals forces. In these cases, gel formation can be reversed (Cerchiara et al., 2002; Berger et al., 2004; Boucard et al., 2005). However, physically cross-linked gels could present some drawbacks; i.e., weak mechanical strength, uncontrolled gel pore size and fast dissolution kinetics (Dash et al., 2011). Improvements of mechanical properties can be obtained by permanent hydrogel networks, using covalent bonding between polymeric chains. In particular, the presence of –NH_2_ and –OH groups on CS chains offers the possibility to create different linkages, such as amide and ester bonding, as well as Schiff base formation (Croisier and Jérôme, 2013). The production of chemically cross-linked gels is achieved by mixing chitosan aqueous solutions with cross-linkers or charged polymers, under specific conditions of pH and temperature (López-León et al., 2005; Liang et al., 2009; Cui et al., 2014; Pellá et al., 2018). Glutaraldehyde (GTA), diglycidyl ether, diisocyanate, and diacrylate, are generally used for this purpose (Hoare and Kohane, 2008). However, their use in pharmaceutical and biomedical applications is restricted, since these cross-linking agents can deactivate or limit drug efficiency and can be cytotoxic for cells (Zeiger et al., 2005; Takigawa and Endo, 2006). In order to overcome these limitations, Takeshita et al. (2021) synthesized chitosan aerogels using a green technology that avoided the use of harmful chemicals, such as aldehyde cross-linkers. In particular, CS aerogels were produced by urea-induced gelation. Urea is an industrial reagent that is widely used due to its low cost and low toxicity. In this study, urea-induced gelation was followed by ethanol exchange and SC-CO_2_ drying. However, a drastic shrinkage was observed for CS samples at each value of urea concentration. Also genipin, that is a natural chemical compound ables to bind amino groups between amino molecules, can be used to produce a cross-linked CS at good mechanical and degradative properties (Dimida et al., 2015). CS hydrogels can be also prepared by increasing pH of the acidic polymer solutions. In this way, a sol-gel transition is promoted, due to hydrophobic interactions (Moura et al., 2007; Tabernero et al., 2020). Pereira et al. (2020) synthesized CS microspheres loaded with silver (Ag) nanoparticles, through pH inversion mechanism, to be used as bactericidal agent. The results revealed that these systems were effective against Gram-positive and Gram-negative microorganisms. The Ag-loaded CS microspheres were also tested as drug delivery systems, and ibuprofen was used as model drug. The addition of Ag nanoparticles into polymeric matrix promoted a significant delay of ibuprofen release that was extended up to 6 h, with respect to CS-only based microparticles. Gómez et al. (2018) fabricated colloidal suspensions of CS/chondroitin that were subsequently freeze dried to obtain lyophilized nanocomplexes. These cryogels were loaded with a garlic extract and tested against *Staphylococcus aureus* (*S. aureus*), a pathogenic microorganism in chronic skin lesions. The dried gels showed a non-homogeneous morphology consisting of fibers and sheets. The introduction of the extract led to an increase of pristine gel hardness and to an enhanced antibacterial action against *S. aureus*. Obaidat et al. (2015) prepared CS-based aerogel carriers using two different technologies, i.e., SC-CO_2_ assisted drying and freeze drying. Then, these carriers were loaded with salbutamol. SEM analysis revealed several cracks and voids in the freeze dried samples; whereas aerogels preserved a high open porosity and textural properties. The release profile of salbutamol from samples produced by supercritical drying could be considered suitable for pulmonary drug delivery systems; the release from cryogels was instead negatively affected by a low porosity and a low value of surface area. Terzić et al. (2018) prepared CS-based gels and investigated how the drying technique used could affect the drug release system behavior. With the aim of increasing CS hydrophilicity, it was blended with itaconic acid and methacrylic acid. Then, thymol, that represents the main constituent of oregano essential oil and has a strong antibacterial action, was supercritically impregnated into the polymeric matrix. Two different techniques were used for drying: air drying that led to xerogels formation, and SC-CO_2_ drying, for aerogel production. SEM analysis showed that xerogels structure was non-porous; indeed, stresses occurred on the pore walls during the extraction of the solvent, inducing the collapse of the gel native structure. On the other hand, during SC-CO_2_ drying, time resulted the key factor of the process to ensure the desired value of porosity: short processing time led to an incomplete removal of the solvent and, subsequently, a low value of porosity was detected. Due to the high specific surface area, the amount of thymol incorporated into CS aerogel was much higher than the amount loaded into CS xerogel. López-Iglesias et al. (2019) produced CS aerogels loaded with vancomycin to treat infections in chronic wounds. Hydrogel macroparticles were prepared via sol-gel processing and alcogels were subsequently dried. However, xerogels were not able to preserve the intrinsic gel nanoporous structure and high shrinkage values were detected; whereas CS aerogels preserved the overall porosity. A fast release of vancomycin from CS aerogel particles was measured during the first hour and it was followed by a slower release during the next hours.

The initial drug burst effect can be considered a relevant drawback when a sustained drug release is required (Spinks et al., 2006; Aryaei et al., 2014; Xie et al., 2018; Wahba, 2020). To address this issue, CS hydrogels can be covalently cross-linked using UV irradiation or GTA, to obtain an improved mechanical stability (Zeiger et al., 2005; Takigawa and Endo, 2006; Baldino et al., 2015). Baldino et al. (2015) produced CS aerogels by SC-CO_2_ drying. These samples were characterized by a nanofibrous structure, with an average pore size of 100 nm. Moreover, they demonstrated that, thanks to this process, it was possible to obtain a complete removal of GTA from CS gels: the supercritical mixture (CO_2_ + ethanol) showed a high affinity toward GTA, favoring its removal from the samples. For this reason, SC-CO_2_ drying can be considered a promising process to purify chemically cross-linked CS aerogels, to be used for pharmaceutical applications. Mirzaei et al. (2013) prepared CS aerogels cross-linked with GTA for drug delivery. SEM images demonstrated that these xerogels had an average pore size ranging from 100 to 500 μm. Moreover, the swelling trend of these CS xerogels decreased by increasing the amount of cross-linker.

Recently, nanohybrid gels have been used as drug delivery carriers. These pharmaceutical systems are composite materials; they are made up using organic polymers loaded with inorganic nanoparticles. Nanoparticles are supposed to suppress burst drug release behavior, leading to a slower and more continuous release of drugs. Wang et al. (2017) prepared hybrid cryogels of CS, carboxymethyl cellulose (CMC) and graphene oxide (GO), crosslinked by Ca^+2^. These gels were synthesized using an electrostatic self-assembly approach, followed by freeze drying. SEM images showed a cryogel morphology that was mainly characterized by irregular CS-CMC clusters located on GO sheets. These samples were used to investigate the release of 5-fluorouracil (5-FU), a chemotherapeutic agent adopted in the treatment of cancer. GO addition delayed the release of 5-FU and overcame burst release problems associated with CS-based aerogels. Dinu et al. (2016) synthesized CS/clinoptilolite (CPL) biocomposite cryogels by cryogelation. DIC and indomethacin (IDM) were loaded into these cryogels using the solvent evaporation technique. The release profiles of DIC and IDM from CS/CPL composites were pH-dependent, and drug release increased when pH varied from 1.2 to 7.4. Mahanta et al. (2019) realized CS nanohybrid cryogels using two kinds of disk-shaped nanofillers of opposite surface charges, 30B nanoclay (CS-C), negatively charged, and layer double hydroxide (CS-L), positively charged. The antibacterial drug, tetracycline hydrochloride (TC), was used as a model drug to investigate the release kinetics. Nanohybrids exhibited sustained release kinetics in both cases (hydrogel and dried gels). Scaffolds induced a 90, 69, and 56% of drug release in 15 h, from CS, CS-C, and CS-L, respectively; whereas a 58, 40, and 28% of drug release was measured using the respective hydrogels. A critical summary of these papers is reported in Table 1.

**TABLE 1:** Gels of agarose, alginate and chitosan, applied in drug delivery.

References	Materials	Process	Advantages	Disadvantages
Mirzaei et al. (2013)	GTA/CS	SC-CO_2_ drying	Improved mechanical stability of CS upon the addition of GTA	GTA caused negative effects on pepsin activity
García-González et al. (2015)	KET/ALG	SC-CO_2_ drying	Accelerated KET release	pH-sensitive aerogels
Obaidat et al. (2015)	Salbutamol/CS	SC-CO_2_ drying Freeze drying	Aerogel provided good salbutamol release kinetics	Salbutamol release was negatively affected by low porosity values
De Cicco et al. (2016)	ALG/Aminated pectin/Doxycycline	Prilling + SC-CO_2_ drying	Aerogel characterized by open pore structure and high specific surface area	Complex process
Dinu et al. (2016)	CPL/DIC/IDM/CS	Cryogelation + solvent evaporation	The addition of inorganic particles (CPL) improved CS gel stability during drug release	Time-consuming process
Sukhodub et al. (2018)	HAp/ALG/CHX	Air drying Freeze drying	Air drying is an energy-save process	During air drying, only a small amount of CHX was incorporated into the scaffolds
Wang et al. (2017)	CS/CMC/GO/5-FU	Electrostatic self-assembly approach + SC-CO_2_ drying	Burst effect associated to CS-based aerogel was eliminated after GO addition	Time-consuming process; Irregular morphology
Gómez et al. (2018)	CS/Chondroitin/ *S. aureus*	Freeze-drying of colloidal suspensions	Cryogels with enhanced antibacterial action against *S. aureus*	Time-consuming process
Mustapa et al. (2018)	*C. nutans*/ALG	SC-CO_2_ drying + SC-CO_2_ impregnation	The same amount of drug was incorporated into the gel using supercritical impregnation instead of organic solvents	Time-consuming process
Athamneh et al. (2019)	Sodium Alginate/Hyaluronic acid	Emulsion gelation + SC-CO_2_ drying	High specific surface area	No experiments *in vitro* were performed
Franco and De Marco (2020)	NIM/KET/DIC/MSTR/CAALG	SC-CO_2_ drying + SC-CO_2_ impregnation	CAALG aerogel promoted a controlled release of non-steroidal anti-inflammatory drugs	Not all the solubilised drug in SC-CO_2_ was absorbed onto the aerogel, and the non-absorbed drug can precipitate in form of nanoparticles
López-Iglesias et al. (2019)	Vancomycin/CS	SC-CO_2_ drying Freeze drying Air evaporation	Overall aerogel porosity preserved during the supercritical drying	Cryogels and xerogels showed a condense structure; Burst effect was detected during the drug release from aerogel
Kim et al. (2019)	βCD/ETAGR/BSA/DOX	Freeze drying	AGR derivatives allowed the production of a DD system	ETAGR had a lower cross-linking density than unmodified AGR and, for this reason, burst effect was detected during the drug release test
Lynam et al. (2015)	Sucrose-AGR/Proteins	SC-CO_2_ drying	Sucrose modified hydrogels were characterized by smaller and more uniform pore size	Sucrose particles could be present on the gel
Mahanta et al. (2019)	CS-C/CS-L/TC	Freeze drying	The addition of nanofillers provided a less collapsible pore structure; Nanocomposite exhibited a sustained drug release	CS aerogel showed a collapsible cell structure
Yuan et al. (2018)	KGM/AGR/Ciprfloxacin	Freeze drying	Drug load efficiency and sustained release capacity of AGR hydrogels were enhanced by KGM incorporation	AGR hydrogels showed a significant burst effect
Witzler et al. (2019)	Amoxicillin/AGR-coated HAp	Freeze drying SC-CO_2_ drying	Supercritically dried samples did not exhibit large pores and seemed to be very homogeneous; Composite materials slowed down drug release for both water-soluble drugs	AGR scaffolds exhibited an initial burst release
Pereira et al. (2020)	Ibuprofen/Ag/CS	pH inversion + under vacuum evaporation	A sustained release of ibuprofen was ensured	Epichlorohydrin was used as chemical cross-linker
Trucillo et al. (2020)	Ampicillin-loaded liposomes/CAALG	SuperLip + SC-CO_2_ drying	A more sustained ampicillin release was ensured using the meta-carrier	Part of the liposomes was lost in the solvent used for solvent-exchange
Takeshita et al. (2021)	Urea/CS	Urea-induced gelation + SC-CO_2_ drying	Toxic chemical cross-linkers were avoided	Low values of urea concentration led to a drastic shrinkage of the final sample

## Alginate-Based Gels

Alginate is a naturally occurring anionic polymer, typically obtained from brown seaweed, and has been extensively used for pharmaceutical applications, thanks to its biocompatibility, low toxicity, low cost and easy gelation (García-González et al., 2015; Pantić et al., 2016; Athamneh et al., 2019; Lovskaya and Menshutina, 2020). ALG hydrogels formation can be induced by different cations, such as: H^+^, Ca^+2^, Ba^+2^, Cu^+2^, Sr^+2^, Zn^+2^, Mn^+2^, Fe^+2^, Al^+3^, and Fe^+3^ (Cao et al., 2020). Indeed, the presence of negatively charged ions in ALG molecules can lead to the formation of polyelectrolyte complexes, because they give the possibility to bind positively charged ions (Hu et al., 2021). Ca^+2^ is the most used divalent cation to induce alginate gelation, since it shows a high affinity toward the bio-polymer guluronate (G) blocks (Hu et al., 2021). Reynolds and Enquist (1973) proposed, for the first time, the theory of Ca^+2^ induced ALG gelation mechanism, defining the gel structure as an egg-box model. In this structure, Ca^+2^ coordinates with six oxygen atoms of two neighboring G units and one to three oxygen atoms of H_2_O to form a stable structure. However, depending on the final application, different kind of ions can be used: e.g., Ba-ALG gels have been widely used in nanomedicine and Sr-ALG gels show a great potential for tissue regeneration, since they can enhance cell proliferation (Hu et al., 2021). One critical drawback of ionically cross-linked ALG gels is the limited long-term stability in physiological conditions, because these gels can easily dissolve due to the release of divalent ions into the surrounding media, as a consequence of exchange reactions with monovalent cations (Waldman et al., 1998; Qin, 2004, 2005; Santos Miranda et al., 2006).

ALG hydrogels similarity to the extracellular matrices of living tissues allows wide applications in the delivery of small chemical drugs and proteins (Rowley et al., 1999; Augst et al., 2006; Bidarra et al., 2014). Athamneh et al. (2019) realized aerogel microspheres based on sodium alginate and hyaluronic acid for pulmonary drug delivery. Emulsion gelation was combined with SC-CO_2_ gel drying and, at the end, aerogels, at high specific surface area and good aerodynamic properties, were obtained. García-González et al. (2015) investigated the release kinetics of ketoprofen (KET) from ALG-based aerogels. These authors found that ALG aerogels accelerated KET release at simulated gastric pH conditions. Moreover, it was noted that KET release was mainly governed by a Fickian diffusion mechanism. Sukhodub et al. (2018) synthesized a hydroxyapatite-alginate (HAp)-ALG nanostructured composite for the controlled release of chlorexidine (CHX). The dried samples were obtained by hydrogel drying at 37°C in warm ambient and by freeze drying for 24 h. The densest morphology corresponded to the composite xerogel dried at 37°C; whereas the freeze dried sample had a porous morphology whose homogeneity was slightly improved after HAp addition. Increasing the amount of ALG in the composite gels led to an increase in the volume of adsorbed and released CHX, and the release time also increased from 24 to 72 h. Trucillo et al. (2020) produced a meta-carrier; namely, a carrier entrapped inside another carrier, formed by a liposome loaded aerogel. The antibiotic (ampicillin) was encapsulated into the liposomes, produced by a supercritical assisted liposomes formation (SuperLip) technique. SC-CO_2_ drying was selected to produce ALG aerogels loaded with ampicillin loaded liposomes. The structures obtained in the case of water exchange with ethanol and acetone were characterized by different morphologies; in particular, the structures obtained in the first case showed nanofibers and open pores, whereas the other ones were uniformly nanoporous. Drug release tests demonstrated that ampicillin release time from these meta-carriers was about twice than its release time from liposomes alone.

Franco and De Marco (2020) used SC-CO_2_ adsorption to incorporate three non-steroidal anti-inflammatory drugs (NSAIDs), nimesulide (NIM), (KET) and diclofenac sodium (DIC), into maize starch (MSTR) and calcium alginate (CAALG) aerogels. For each NSAID, it can be noted that the amount of drug adsorbed in CAALG was generally higher than the amount loaded in MSTR. Both aerogels were formed by a microporous structure, preserved after supercritical adsorption. The dissolution tests revealed that the adsorption into MSTR allowed a faster release of NSAIDs than pure crystalline drugs; whereas CAALG promoted a controlled release of NSAIDs. Gonçalves et al. (2016) realized ALG-based hybrid aerogels in form of particles to be used as mucosal drug delivery systems. Gel drying and KET loading were performed using SC-CO_2_. All ALG-based macroparticles showed high specific surface area and large pore volume. Moreover, these formulations were able to provide a slower release of KET in comparison with the pure one. De Cicco et al. (2016) realized aerogel formulations based on ALG and amidated pectin, in form of core-shell microparticles, that were dried using SC-CO_2_. At the end of the process, these samples were characterized by an open pore structure and high specific surface area. These polymeric aerogels were loaded with doxycycline and the results demonstrated that drug release was affected by pectin and ALG amount. Moreover, doxycycline release kinetics was mainly governed by swelling matrix and erosion phenomena. Mustapa et al. (2018) produced ALG hydrogels that were supercritically dried. Plant extracts of *Clinacanthus nutans (C. nutans)* were impregnated into ALG aerogels via SC-CO_2_ assisted impregnation. *C. nutans*-50 extract was released faster from ALG when was impregnated using supercritical conditions. A critical summary of these papers is reported in Table 1.

## Agarose-Based Gels

Agarose is currently used in various research fields including food, DD, DNA electrophoresis, and tissue engineering, owing to its thermo-reversible gel forming ability (Sakai et al., 2007; Gu et al., 2017).

In order to create an AGR gel, heating and cooling processes are involved, and gelation occurs at high temperatures, making difficult to load heat-sensitive drugs. Therefore, researchers have developed AGR gels with low gelling temperatures by modification through acetylation (García-Ruiz et al., 2001), alkylation, alkenylation, acylation, and oxyalkylation (Zhang et al., 2018). The introduction of functional groups hinders the formation of the helicoidal structure at low temperatures, thereby lowering the gelling temperature of AGR (Forget et al., 2015). Moreover, for the development of more innovative AGR-based materials, AGR should comprise special functional groups, such as tosyl or amine moieties (Gericke and Heinze, 2015).

The diffusion characteristics of various substances, including drugs, from AGR gels, are related to the rheological properties of the gels (Normand et al., 2000; Kim et al., 2019). Therefore, AGR derivatives with low gelation temperatures can deliver the substance quickly because the double helicoidal structure during gelation is reduced and, subsequently, the storage modulus is lowered. For this reason, there is a limit in developing effective DD systems that can control the release rates using AGR derivatives with low gelation temperatures (Kim et al., 2019).

Kim et al. (2019) described the introduction of β-cyclodextrin (βCD) into an ethylenediamine-modified agarose (ETAGR) for the development of AGR at low gelling temperatures. The modified gels were prepared by freeze drying and used for both bovine serum albumin (BSA) and doxorubicin (DOX) release. The section of non-functionalized AGR gel was characterized by a non-uniform distribution of pore sizes. The release profiles showed that increasing the cross-linking density of the gel or increasing AGR concentration, the diffusion rate and, subsequently, the release kinetics of BSA, slowed down. Since CFAs had lower cross-linking density than AGR, due to the presence of ethylenediamine groups, BSA was released within a few hours with a non-negligible initial burst effect. Lynam et al. (2015) developed AGR scaffolds used for the controlled release of proteins, for nerve repair. Solvogels were supercritically dried after the replacement of water in AGR hydrogel with ethanol. Pores of 0.10 μm were identified; moreover, SEM images showed a homogeneous and nanoporous morphology. Witzler et al. (2019) investigated the release behavior from AGR-coated Hap, using two model drugs, i.e., adenosine 5′-triphosphate (ATP) and suramin. A prolonged release over 4 days was found for both samples (cryogel and aerogel). However, freeze dried samples exhibited large pores in the range of several hundred micrometers. This was due to the growth of ice crystals during the freezing process. In contrast to the freeze dried samples, the SC-CO_2_ dried ones were characterized by a higher specific surface area. Yuan et al. (2018) produced a polymeric blend with konjac glucomannan (KGM) and AGR, for ciprofloxacin release, using freeze drying. KGM is a natural polysaccharide found in the tuber of *Amorphophallus konjac*. The addition of KGM determined a clear effect on the internal morphology of AGR gels: the increase in KGM led to a more compact internal structure with smaller pores. The release results demonstrated that encapsulation, drug loading efficiencies, and sustained release capacity of AGR cryogels, were enhanced by the incorporation of KGM. However, more than 95% of ciprofloxacin was released in the first half hour, because most of the drug was localized on the surface of the polymeric matrix. A critical summary of these papers is reported in Table 1.

## Conclusion

In this short review, the main production techniques of drug delivery systems, based on natural, biocompatible and biodegradable polymers, were analyzed. Bio-based aerogels are supposed to be promising candidates as drug carriers, thanks to the native overall nanoporosity and the high specific surface area. These features allow to reach a high drug loading and to obtain a sustained drug release over time.

Freeze drying and air evaporation are the most consolidate and frequently used drying techniques to produce these bio-polymeric systems. However, they can lead to dense and/or not homogeneous final gel structures; therefore, alternative drying techniques should be selected to preserve these relevant characteristics for drug release. Supercritical CO_2_ assisted drying can overcome these drawbacks, allowing to produce nanostructured bio-aerogels, that maintain the starting gel volume and shape. These aerogel properties are preserved when (i) water/solvent exchange step is carefully performed by selecting the opportune organic solvent and a slow exchange rate, and (ii) the process operative conditions guarantee the formation of a supercritical mixture (CO_2_ + organic solvent) at negligible surface tension. Moreover, SC-CO_2_ assisted drying can also remove cross-linking agent residues from the aerogels, making these advanced bio-carriers safe and suitable for controlled drug delivery applications.

## Author Contributions

MG and LB: conceptualization. MG: writing—original draft preparation. LB: writing—review and editing. ER: supervision. All authors contributed to manuscript revision, read, and approved the submitted version.

## Conflict of Interest

The authors declare that the research was conducted in the absence of any commercial or financial relationships that could be construed as a potential conflict of interest.

## Abbreviations

Ag: silver
AGR: agarose
ALG: alginate
ATP: adenosine 5′-triphosphate
BSA: bovine serum albumin
CAALG: calcium alginate
CPT: camptothecin
β CD: β-cyclodextrin
CMC: carboxymethyl cellulose
CHX: chlorexidine
CPL: clinoptilolite
CS: chitosan
CS-C: nanoclay-loaded chitosan
CS-L: layer double hydroxide-loaded chitosan
DIC: diclofenac sodium
DOX: doxorubicin
ETAGR: ethylenediamine-modified agarose
5-FU: 5-fluorouracil
GO: graphene oxide
GTA: gluteraldehyde
G: guluronate
HAp: hydroxyapatite
IDM: indomethacin
KET: ketoprofen
KGM: konjac glucomannan
MSTR: maize starch
NIM: nimesulide
NSAIDs: non-steroidal anti-inflammatory drugs
PEG: polyethyleneglycol
PVDF-HFP: poly(vinylidene fluoride-hexafluoropropylene)
S. aureus: staphylococcus aureus
SC-CO_2_: supercritical CO_2_
SuperLip: supercritical assisted liposomes formation
TC: tetracycline hydrochloride.
